# Amino-Modified ZIF-90 for Effective Adsorption of Au(III) in Environmental Water

**DOI:** 10.3390/molecules30081826

**Published:** 2025-04-18

**Authors:** Na Zhou, Xueli Wu, Shaoxia Wang, Jianfei Qu, Yang Tan, Chuanlei Luan, Xiuli Yin, Xuran Wu, Xuming Zhuang

**Affiliations:** 1 Yantai Institute of Coastal Zone Research, Chinese Academy of Sciences, Yantai 264003, China; nzhou@yic.ac.cn (N.Z.); xlwu@yic.ac.cn (X.W.); ytan@yic.ac.cn (Y.T.); clluan@yic.ac.cn (C.L.); xlyin@yic.ac.cn (X.Y.); 2 School of Chemistry and Chemical Engineering, Yantai University, Yantai 264005, China; 18865557620@163.com (S.W.); 17860398700@163.com (J.Q.); 201001001732@ytu.edu.cn (X.W.)

**Keywords:** amino, ZIF-90, gold ions, adsorption

## Abstract

In this work, amino-modified ZIF-90 (NH_2_-ZIF-90) was prepared by using butylamine as a modifier, and its effectiveness in adsorbing Au(III) from environmental samples was investigated. The morphology and structure of NH_2_-ZIF-90 were analyzed via SEM, XRD, FT-IR, and XPS. Optimal adsorption occurred after 12 h of shaking in a pH = 5 aqueous solution with 2 mg mL^−1^ NH_2_-ZIF-90. The adsorption kinetics conformed to a pseudo-second-order model, and the equilibrium data fit the Freundlich isotherm model well. Finally, NH_2_-ZIF-90 was successfully used in lake water and tap water samples for Au(III) adsorption, with recovery rates ranging from 81.0% to 93.3%. This study presents a novel approach for addressing Au(III) adsorption challenges.

## 1. Introduction

Advancements in science and technology have led to the emergence of many new, high-performance materials, with metal–organic frameworks (MOFs) gaining significant research interest in recent years due to their unique advantages. MOFs, a class of inorganic–organic porous materials, were first proposed in 1995 and have since developed rapidly, gaining much traction. Their high specific surface area, exceptional adsorption performance, thermal and mechanical stability, and modifiability make them versatile for applications in sensing, gas adsorption, catalysis, drug loading, and other fields [1]. Several major MOF series, including isoreticular metal organic frameworks (IRMOFs), zeolitic imidazolate frameworks (ZIFs), materials of Institute Lavoisier frameworks (MILs), and porous coordination frameworks (PCNs), have been identified and studied [2].

As a subfamily of MOFs, ZIFs are similar in structure to silicate. Traditional methods of synthesizing ZIFs primarily include solvothermal and hydrothermal approaches. More recently, ultrasonic chemical, mechanochemical, and electrochemical synthesis methods have been developed [3]. Like other MOFs, ZIFs exhibit excellent chemical stability, large specific surface areas, and adjustable pore sizes, making them highly attractive for applications in the field of metal ion adsorption [4]. Zhao et al. [5] investigated the adsorption behavior and competitive adsorption characteristics of ZIF-8 for heavy metal ions Cu^2+^, Ca^2+^, Ni^2+^, and Co^2+^, analyzing for adsorption kinetics. Their findings revealed that ZIF-8 exhibits strong selectivity for Cu^2+^. Huang et al. [6] studied ZIF-8 and ZIF-67 for adsorption of Pb^2+^ and Cu^2+^ in wastewater. Their results demonstrated that both ZIFs have significantly higher adsorption capacities for Pb^2+^ and Cu^2+^ compared to other porous materials, establishing them as excellent candidates for removing heavy metal ions from wastewater.

Amino-functionalized ZIFs offer enhanced properties compared to their non-functionalized counterparts, making them promising materials for various applications [7]. The choice of amino-functionalized ZIF depends on the specific application, with considerations such as adsorption capacity, catalytic activity, and the potential for further functionalization playing key roles in material selection. Compounds such as ZIF-90-NH_2_ offer additional sites for further chemical modification, making them versatile materials for various applications [8]. The stability of amino-functionalized ZIFs can vary depending on the nature of the amino group and the synthesis conditions. Generally, they retain the high thermal and chemical stability characteristic of ZIFs; the introduction of amino groups may affect their stability under certain conditions.

Gold compounds are the most toxic of the gold subgroup, as are silver and copper. Medically, for gold compounds, it is found that their excretion is very slow, only 20% in two weeks [9]. It can accumulate in the tissues of living organisms, a process known as bioaccumulation. This can lead to the concentration of the metal increasing as it moves up the food chain, potentially affecting top predators, including humans [10]. Furthermore, the presence of Au(III) can degrade the quality of water, making it unsuitable for drinking, irrigation, or industrial use. Even at low concentrations, it can disrupt the balance of aquatic environments, harming fish, plants, and microorganisms [11]. Removing these contaminants ensures the water remains safe and usable for environmentally safe purposes. Overall, the removal of Au(III) from water is crucial for protecting the environment and human health and ensuring the sustainable use of valuable resources.

**Figure 1:**
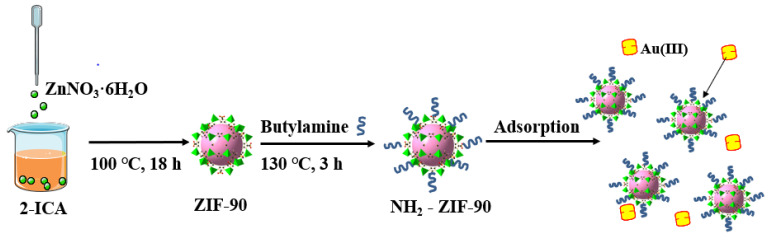
Schematic diagram of NH_2_-ZIF-90 preparation and adsorption process of Au(III).

Many studies have reported the use of ZIF-90 in gas adsorption and separation; however, its potential for metal ion adsorption, particularly for the precious metal Au(III), remains underexplored [12,13]. Notably, ZIF-90 contains aldehyde functional groups, which can be functionally modified for improved adsorption performance without compromising its framework [14,15]. Mei et. al. [11] provides a strategy by designing a hybrid MOF-on-MOF heterostructure for the detection and extraction of gold. The constructed hybrid material exhibits good stability, fast response time, as well as high sensitivity for the detection of Au^3+^. Moreover, the adsorbent showed good selectivity and a high removal rate for gold in actual leaching solutions of CPU-1 and CPU-2. Naghshbandi et. al. [16] used zeolitic imidazolate framework-67 (ZIF-67) functionalized with graphene quantum dots, which was prepared and utilized for the reduction of Au(III) and stabilization of Au nanoparticles. ZIF-67 was utilized as a support for the stabilization of Au NPs, in which the synergetic effect between ZIF-67 and Au demonstrated potential for catalytic application. In this work, ZIF-90 was synthesized via a solvothermal method, chemically modified via a covalent modification method, and the resulting amino-modified ZIF-90 (NH_2_-ZIF-90) was subjected to an Au(III) adsorption study (Figure 1), which yielded satisfactory results.

## 2. Results and Discussion

### 2.1. Characterizations of NH_2_-ZIF-90

**Figure 2:**
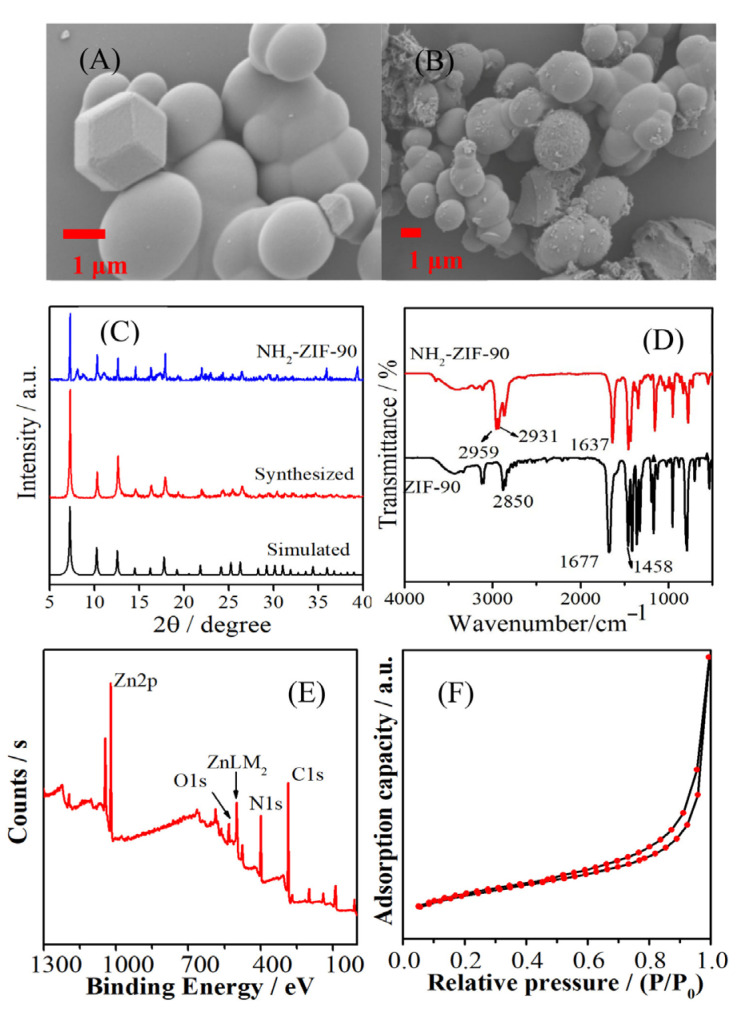
SEM images of (**A**) ZIF-90, (**B**) NH_2_-ZIF-90. (**C**) XRD patterns of simulated ZIF-90, synthesized ZIF-90, and NH_2_-ZIF-90. (**D**) FT-IR spectra of ZIF-90 and NH_2_-ZIF-90. (**E**) XPS survey spectrum. (**F**) N_2_ adsorption–desorption isotherm of NH_2_-ZIF-90.

Figure 2 shows that the ZIF-90 synthesized via the solvothermal method (Figure 2A) has a predominantly spherical morphology, with good crystallinity with diameter 1.6 ± 0.6 μm. After modification with amino groups (NH_2_-ZIF-90, Figure 2B), some ZIF-90 surfaces became slightly rough, but the overall morphology and size characteristics remained unchanged. From Figure S1, the EDS and element mapping images revealed that the NH_2_-ZIF-90 is composed of the desired elements and that the related elements were uniformly distributed, indicating that the NH_2_-ZIF-90 was successfully synthesized, as expected.

Figure 2C shows the XRD spectrum of the material. The synthesized ZIF-90 appeared at 2θ = 7.24°, 10.3°, 12.7°, 14.2°, 16.3°, 17.9°, 21.9°, 24.4°, 26.6°, 29.5°, 30.4°, and 32.1°, with characteristic peaks corresponding to (011), (200), (112), (002), (013), (222), (114), (233), (134), (044), (244), and (235), respectively. The crystal plane is consistent with the XRD spectrum simulated by the ZIF-90 crystallography database and prior literature reports, confirming that the synthesized ZIF-90 has good crystallinity [17]. After amino modification, the resulting XRD spectrum of NH_2_-ZIF-90 shows a small number of impurity peaks, but it remains largely consistent with the unmodified spectrum. This suggests that the framework structure of ZIF-90 is effectively preserved after heating and refluxing. This result also indicates that ZIF-90 retains both thermal and chemical stability, allowing for covalent functionalization under appropriate reaction conditions [18].

The FT-IR spectrum of ZIF-90 in Figure 2D shows that the characteristic peaks of C=O and C–H of the aldehyde group in the organic ligand appear at 1677 and 2850 cm^−1^ [19]. The characteristic peak at 1458 cm^−1^ belongs to the C–H stretching vibration in the imidazole ring, and the peak at 1170 cm^−1^ is the stretching vibration peak of C–N [20]. The peak positions are basically consistent with existing reports in the literature, which verifies that the synthesis of ZIF-90 was successful. After modification, the aldehyde peak at 1677 cm^−1^ shifted to 1638 cm^−1^. The reason is that the –NH_2_ groups of butylamine and the –CHO groups of ZIF-90 undergo a Schiff base condensation reaction [19]. In the reaction, the H atom of the amino group is first transferred to the aldehyde group to form a C–N single bond. After dehydration, C=N is generated, so its characteristic peak appears at 1638 cm^−1^. In addition, anti-symmetric stretching vibration peaks of –CH_3_ and –CH_2_ of butylamine appeared at 2959 and 2931 cm^−1^, indicating that butylamine was successfully bonded onto the material ZIF-90.

The XPS spectrum of NH_2_-ZIF-90 shown in Figure 2E reveals the presence of four elements: C, N, O, and Zn, with respective contents of 57.67%, 31.73%, 6.33%, and 4.27%. The characteristic peaks at 531.3, 398.9, and 284.8 eV belong to O1s, N1s, and C1s, respectively. In addition, the Zn2p signal peaks appear at 1021.7 and 1044.7 eV, and the signal peak of ZnLM_2_ appearing at 499.0 eV was generated by the Auger electron of Zn [21]. The peak position of each element in NH_2_-ZIF-90 is consistent with the relevant literature reports, indicating that butylamine-modified ZIF-90 maintains its original skeleton structure [22].

The N_2_ adsorption–desorption isotherm was used to determine the porosity of the material and obtain basic information such as the specific surface area and pore size. Figure 2F shows the N_2_ adsorption–desorption isotherm of NH_2_-ZIF-90 at low temperature. In the low-pressure area, the adsorption amount increases gradually. Hysteresis occurs after P/P_0_ > 0.6, indicating that NH_2_-ZIF-90 has a porous structure. The specific surface area of NH_2_-ZIF-90 was measured to be 5.28 m^2^ g^−1^, the pore volume was 0.0208 cm^3^ g^−1^, and the pore diameter was 8.31 nm.

### 2.2. Effect of pH, Adsorbent Dosage, and Time on Au(III) Adsorption

**Figure 3:**
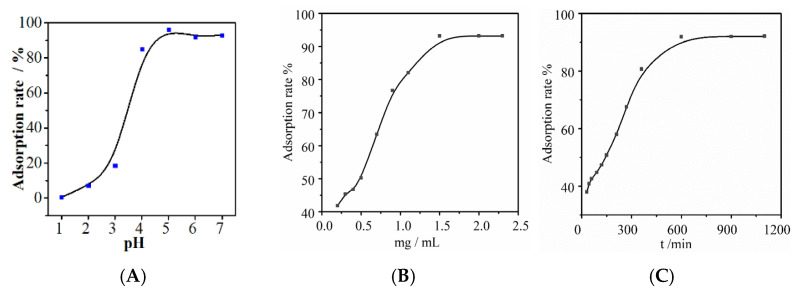
Effect of (**A**) pH, (**B**) the addition amount, and (**C**) adsorption time on the adsorption of Au(III) by NH_2_-ZIF-90.

The pH of a solution affects both the form of metal ions and the surface charge of the adsorbent [23]. To investigate the effects of pH on the adsorption of Au(III) by NH_2_-ZIF-90, experiments were conducted by varying pH 1~7, adjusted by steps of 0.1 M HCl and NaOH (pH values greater than 7 were excluded to avoid gold ion precipitation). As shown in Figure 3A, the adsorption capacity of NH_2_-ZIF-90 for Au(III) gradually increases as the pH rises and eventually stabilizes; the reason is that the greater the acidity is, the greater the degree of damage to the structure of the NH_2_-ZIF-90 and the lower the adsorption effect. Moreover, the higher the pH is, the lower the concentration of H^+^ in the solution, which decreases the competition between H^+^ and Au(III) for adsorption active sites, improving the adsorption effect. In order to minimize the hydrolysis and precipitation of Au(III) and ensure the high adsorption performance of NH_2_-ZIF-90, pH = 5 was selected for subsequent experiments.

The experimental results of the effect of NH_2_-ZIF-90 dosages on the adsorption of Au(III) are shown in Figure 3B. Testing was carried out with conditions of pH = 5, Au(III) concentration of 5 μg mL^−1^, adsorption time of 10 h, and adsorption temperature of 25 °C. As the amount of NH_2_-ZIF-90 increases, the number of available adsorption sites also increases, leading to a gradual increase in adsorption. However, since the concentration of Au(III) in the solution is limited, further addition of NH_2_-ZIF-90 tends to equilibrium. Therefore, the optimal dosage of NH_2_-ZIF-90 is 2 mg mL^−1^.

To determine whether adsorption time affects the adsorption of Au(III) by NH_2_-ZIF-90, an experiment with adsorption time as the sole variable was conducted next. The experimental results shown in Figure 3C indicate that with a fixed amount of NH_2_-ZIF-90, the adsorption rate of NH_2_-ZIF-90 to Au(III) is positively correlated with the adsorption time until equilibrium is reached. This trend is because the number of adsorption sites for Au(III) in the solution is limited. With increasing adsorption time, the adsorption sites of Au(III) gradually become saturated and lose their adsorption capacity, leading to a plateau in the adsorption rate. The time of 12 h was selected as the best adsorption time for subsequent experiments.

### 2.3. Adsorption Kinetics Studies

Adsorption kinetics studies are mainly used to describe the speed with which an adsorbent adsorbs a solute and to clarify the adsorption mechanism. In this study, four kinetics models were employed to evaluate the mechanism of Au(III) adsorption by NH_2_-ZIF-90: pseudo-first-order, pseudo-second-order, intraparticle diffusion, and Elovich kinetic models. The equations for the four kinetics models are as follows:

Pseudo-first-order kinetic model:

(1) $$Q_{t}=Q_{e}\left(1-e^{-K_{1}t}\right)$$

Pseudo-second-order kinetic model:

(2) $$Q_{t}=\frac{K_{2}Q_{e}^{2}t}{1+K_{2}Q_{e}t}$$

Intraparticle diffusion model:

(3) $${\;Q}_{t}=K_{\mathrm{id}}t^{0.5}+C$$

Elovich kinetic model:

(4) $${\;Q}_{t}=\frac{1}{\beta }\ln(1+\alpha \;\beta \;t)$$

where *K*_1_ (min^−1^), *K*_2_ (g mg^−1^ min^−1^), and *K*_id_ (g mg^−1^ min^−0.5^) are the pseudo-first-order, pseudo-second-order, and intraparticle diffusion rate constants, respectively. *Q*_e_ and *Q*_t_ (mg g^−1^) are the amounts of Au(III) adsorbed at equilibrium and at time *t*, respectively, and *t* (min) is the adsorption time. *α* (g mg^−1^ min^−1^) and *β* (g mg^−1^) are the initial adsorption rate and a constant related to surface coverage and activation energy, respectively.

**Figure 4:**
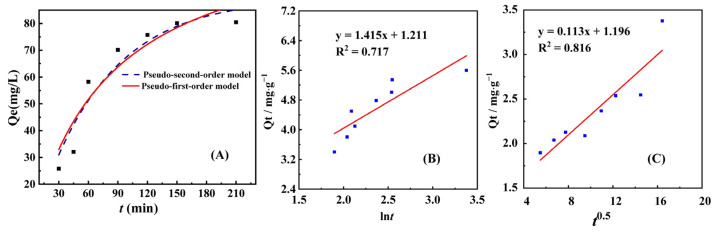
(**A**) Pseudo-first-order kinetic model (red curve), pseudo-second-order kinetic model (blue curve), (**B**) Elovich kinetic model, and (**C**) intraparticle diffusion for the adsorption of Au(III) by NH_2_-ZIF-90.

In order to further elucidate the adsorption kinetics process, two broad kinetic models, pseudo-first-order kinetic model and pseudo-second-order kinetic model, were used for nonlinear regression analysis. In order to visually demonstrate the results of nonlinear regression analysis, we accurately compared and plotted the fitted curve obtained from the analysis with the original experimental data, and presented the relevant results in the form of Figure 4A. By comparison, it can be found that there are certain differences in the fitting effect of different models on experimental data. The pseudo-first-order kinetic model may exhibit good fitting performance in the early stage of adsorption. The relevant kinetic parameters are summarized in Table 1 and Table 2. Based on the fitted correlation coefficient (R^2^) values, the pseudo-first-order kinetic model (R^2^ = 0.996) is more suitable for describing the adsorption process, indicating that the adsorption behavior is likely governed by physical diffusion or surface-based mechanisms rather than chemisorption-driven kinetics associated with covalent bond formation. The intraparticle diffusion model fitting reveals that the plot of *Q_t_* vs. *t*^0.5^ forms a linear equation without passing through the origin; this indicates that internal diffusion is not the sole rate-controlling step, and other adsorption mechanisms contribute to the process [24].

### 2.4. Adsorption Isotherm Studies

The equilibrium adsorption isotherm is important in adsorption systems for evaluating the metal ion uptake mechanism and capacity [25]. The Langmuir isotherm (Equation (5)) and Freundlich isotherm (Equation (6)) were applied to fit the experimental data.

(5) $$\frac{C_{e}}{Q_{e}}=\frac{1}{K_{L\;}Q_{m}}+\frac{C_{e}}{Q_{m}}$$

(6) $$\ln Q_{e}=\ln K_{F}+\frac{1}{n}\;\ln C_{e}$$

Here, *Q*_m_ (mg g^−1^) is the monolayer saturated adsorption amount, and *K*_L_ (L mg^−1^) is the Langmuir adsorption constant. *K*_F_ and n are the Freundlich constants related to adsorption capacity and adsorption strength, respectively.

The adsorption isotherm of Au(III) by NH2-ZIF-90 was simulated using both the Langmuir and Freundlich isotherm models to investigate the interaction mechanism [26]. As evidenced by the results presented in Figure S2 and Table S1, the Langmuir adsorption isotherm demonstrates a higher degree of congruence (R^2^ = 0.951) describing this adsorption process compared to the Freundlich model. This alignment with the Langmuir model indicates that the adsorption predominantly occurs through a monolayer surface saturation mechanism rather than multilayer adsorption [27], suggesting chemical interactions between Au(III) ions and the homogeneous active sites on the NH_2_-ZIF-90 adsorbent.

### 2.5. Adsorption Thermodynamics Studies

To explore the internal energy changes in NH_2_-ZIF-90 during the adsorption process, the thermodynamic characteristics were studied to clarify the adsorption mechanism [28]. The thermodynamic parameters were evaluated using the following equations:

(7) $$\ln K_{c}=-\frac{\Delta H}{RT}+\frac{\Delta S}{R}$$

(8) $$\Delta K_{c}=\frac{Q_{e}}{C_{e}}$$

(9) $$\Delta G=-\mathrm{RT}\ln K_{c}$$

where Δ*H* (J mol^−1^), Δ*S* (J mol^−1^ K^−1^), and Δ*G* (J mol^−1^) are the enthalpy, entropy, and Gibbs free energy changes, respectively. *R* is the ideal gas constant (8.314 J mol^−1^ K^−1^), and *T* (K) represents the temperature in Kelvin. *K*_c_ (L g^−1^) is a thermodynamic constant.

Using the data obtained in the temperature range of 288.15 to 328.15 K, the thermodynamic equilibrium constant K_C_ was derived from the Langmuir isotherm parameter K_L_ for rigorous thermodynamic analysis. Table 3 presents data on Δ*H*, Δ*S*, and Δ*G*. The values of Δ*H* and Δ*G* are –37.09 J mol^−1^ and 5.952 J mol^−1^·K^−1^, respectively, indicating that the adsorption of Au(III) by NH_2_-ZIF-90 is exothermic and spontaneous. Δ*S* > 0 indicates that the degree of freedom of the system increases during the adsorption process [29].

### 2.6. Influence of Interference Ions on Adsorption Properties

During the adsorption process, external interfering ions may compete with Au(III) for active sites on NH_2_-ZIF-90, potentially affecting adsorption efficiency. Therefore, the influence of common coexisting metal ions, such as K^+^, Na^+^, Zn^2+^, Cu^2+^, Fe^3+^, Pb^2+^, Mg^2+^, and Cd^2+^, on the adsorption of Au(III) by NH^2^-ZIF-90 was explored. The results show that even in the presence of 20 times the concentrations of K^+^ and Na^+^, and 10 times the concentrations of Zn^2+^, Cu^2+^, Fe^3+^, Pb^2+^, Mg^2+^, and Cd^2+^ (compared to the concentration of Au(III)), NH_2_-ZIF-90 has a positive effect on Au(III). This indicates that the adsorption selectivity of NH_2_-ZIF-90 for Au(III) is excellent.

### 2.7. Adsorption Application of Au(III) to Water Samples

Figure S3 shows the XRD and FTIR spectrum of NH_2_-ZIF-90 after adsorption of Au(III). From Figure S3A, no obvious changes in characteristic peaks occurred, indicating that the crystal structure of the NH_2_-ZIF-90 remained stable during the adsorption process. As shown in Figure S3B, most of the peaks remain unchanged, demonstrating the reliable stability of NH_2_-ZIF-90. The strength and peak position of characteristic peaks at 1638 cm^−1^ are obviously changed, which indicates that the adsorption process of gold ions is coordinated with the Schiff base structure [30]. The regeneration ability and reusability of NH_2_-ZIF-90 were evaluated by performing adsorption–desorption cycles. After each cycle, we measured the nanomaterial’s adsorption capacity and analyzed its structural and chemical properties, comparing the XRD and FTIR results before and after cycling to identify structural changes. The results revealed no significant structural changes, suggesting high stability under the tested conditions. The NH_2_-ZIF-90 maintained 90% of its initial adsorption capacity after 10 cycles, indicating excellent reusability.

Lake water and tap water samples were collected as experimental objects and used to evaluate the performance of NH_2_-ZIF-90 adsorbing Au(III) from environmental samples. The experimental results are shown in Table 4. First, the two collected water samples were left to stand for 24 h to obtain the supernatant. After filtering the supernatant with a 0.45 μm filter membrane, the water samples were processed by a digestion procedure. Under optimal experimental conditions, NH_2_-ZIF-90 was used for the adsorption and desorption of Au(III) in water samples with hydrochloric acid and thiourea as adsorbents. A spike recovery experiment was then conducted, and ICP–AES was used to measure the concentration of Au(III) in the filtrate. The data indicated that NH_2_-ZIF-90 exhibited effective adsorption of Au(III), with a recovery rate after standard addition ranging from 81.0 to 93.3%.

## 3. Materials and Methods

### 3.1. Materials

Imidazole-2-carbaldehyde (C_4_H_4_N_2_O, molecular weight 96.09) was purchased from Aladdin Biochemical Technology Co., Ltd. (Shanghai, China). Zinc nitrate hexahydrate (ZnNO_3_·6H_2_O, molecular weight 297.51) was obtained from Sigma-Aldrich Trading Co., Ltd. (Shanghai, China). N,N-dimethylformamide (C_3_H_7_NO, molecular weight 73.09) was obtained from Sinopharm Group Chemical Reagent Co., Ltd. (Beijing, China). Chemical reagents, including butylamine (C_4_H_11_N, molecular weight 73.14), methanol (CH_3_OH, molecular weight 32.04), and ethanol (C_2_H_5_OH, molecular weight 46.07), were of analytical grade. A standard solution of Au(III) was supplied by the National Steel Materials Testing Center (Beijing, China). All the chemicals used were of analytical reagent grade. All solutions were prepared with ultrapure water (18.2 MΩ cm).

### 3.2. Apparatus

The micromorphology of the samples was analyzed by a scanning electron microscopy (SEM), performed on a JSM-7900F field emission scanning electron microscope at 2 kV (JEOL Ltd., Tokyo, Japan), with energy dispersive spectrometer (EDS) and element mapping images. X-ray diffraction (XRD) patterns were obtained using a Rigaku SmartLab III diffractometer (Rigaku, Cedar Park, TX, USA). Fourier transform infrared spectroscopy (FT-IR) was conducted on an L128-0099 PerkinElmer Spectrometer (Waltham, MA, USA) using KBr pellets. X-ray photoelectron spectroscopy (XPS) was performed using a Thermo ESCALAB 250Xi instrument (Thermo Fisher Scientific, Waltham, MA, USA). The Brunauer–Emmett–Teller (BET) surface area was determined via N_2_ adsorption–desorption at 77 K using a NOVA 3000eN30-20 surface area and pore size analyzer (Anton Paar, Thane, India). Thermogravimetric differential scanning calorimetry (TG–DSC) analysis was carried out on a NETZSCH STA409 PC/PG (NETZSCH, Selb, Germany) at a scan rate of 10 °C min^−1^ under N_2_ flow (30 mL min^−1^). Inductively coupled plasma atomic emission spectroscopy (ICP–AES) was performed on an IRIS Intrepid II (Spectralab Scientific Inc., Markham, ON, Canada).

### 3.3. Synthesis of NH_2_-ZIF-90

To synthesize NH_2_-ZIF-90, 1.92 g of 2-ICA was dissolved in 100 mL of DMF by stirring and heating at 60 °C. Next, 1.49 g of ZnNO_3_·6H_2_O was added as an initiator, and the solution was magnetically stirred at room temperature for 1 h to ensure thorough mixing. The mixed solution was then slowly transferred to an autoclave and left at 100 °C for 18 h to react. After the reaction, the yellow–brown product was centrifugally washed with methanol, and then the product was vacuum dried at 60 °C for 24 h to yield ZIF-90. Then, 0.05 g of ZIF-90 powder was dispersed in 10 mL of ethanol via ultrasonication in a round-bottom flask. A total of 600 μL of butylamine was added to the reaction system, which was then incubated in an oil bath at 130 °C for 3 h. After the reaction period, the obtained product was centrifugally washed with methanol three times and dried to obtain NH_2_-ZIF-90.

### 3.4. Adsorption Studies

A certain amount of NH_2_-ZIF-90 was added to a 50 mL centrifuge tube with 5 μg mL^−1^ Au(III) standard solution. The centrifuge tube was then placed in a constant-temperature water bath shaker for a specific period of time to allow for complete contact between the NH_2_-ZIF-90 and Au(III) standard solution. After adsorption, the NH_2_-ZIF-90 was filtered out, and the residual Au(III) remaining in the filtrate was analyzed and determined via inductively coupled plasma atomic emission spectrometry (ICP–AES), with a blank control prepared for comparison. The effects of pH, shaking time, adsorbent dosage, Au(III) concentration, and temperature on the adsorption effect were investigated. The amount of Au(III) adsorbed onto the SHGNAs (adsorption capacity, *Q*_e_) and the adsorption rate were calculated according to Formulas (10) and (11).

(10) $$\mathrm{Adsorption}\;\mathrm{capacity}\;Q_{e}=\frac{(C_{0}-C_{e})\times V}{m}$$

(11) $$\mathrm{Adsorption}\;\mathrm{rate}(\%)=\frac{\left(C_{0}-C_{e}\right)}{C_{0}}\times 100\%$$

where *Q*_e_ (mg g^−1^) is the adsorption capacity, *C*_0_ (mg L^−1^) is the initial concentration of Au(III), and *C*_e_ is the equilibrium concentration. *V* represents the volume of solution (L), and *m* represents the mass of the adsorbent (g).

## 4. Conclusions

In summary, ZIF-90 was synthesized via the traditional solvothermal method, with 2-ICA as the organic ligand and Zn^2+^ as the inorganic metal ion. The amino group on ZIF-90 was then modified to prepare NH_2_-ZIF-90, which was then applied to environmental water samples to test the adsorption of Au(III). Structural and compositional characterizations of the prepared NH_2_-ZIF-90 were performed. Additionally, the effects of various factors influencing the adsorption of the precious metal ion Au(III) by NH_2_-ZIF-90 were investigated, such as the pH of the solution and the amount of NH_2_-ZIF-90 added. Data fitting revealed the most suitable adsorption isotherm, and the adsorption kinetic model for NH_2_-ZIF-90 adsorption of Au(III) was obtained. Additionally, the thermodynamic properties of the adsorption process were evaluated, and key thermodynamic parameters were calculated. Finally, the prepared NH_2_-ZIF-90 was applied to samples of lake water and tap water to evaluate adsorption of Au(III) in environmental samples. The results show that NH_2_-ZIF-90, as an adsorbent, has important potential application value in metal ion adsorption in the environmental field.

## Figures and Tables

**Table 1:** Pseudo-first-order and pseudo-second-order kinetic parameters for adsorption of Au(III) by NH_2_-ZIF-90.

Pseudo First Order			Pseudo Second Order		
*Q*_e_ (mg g^−1^)	*K*_1_ (min^−1^)	R^2^	*Q*_e_ (mg g^−1^)	*K*_2_ (g mg^−1^ min^−1^)	R^2^
2.764	1.0557	0.996	4.686	0.01411	0.901

**Table 2:** Elovich and intraparticle diffusion kinetic parameters for adsorption of Au(III) by NH_2_-ZIF-90.

Elovich		Intraparticle Diffusion		
*β* (g mg^−1^)	R^2^	*K*_id_ (g mg^−1^·min^−0.5^)	R^2^
0.706	0.717	0.113	0.816

**Table 3:** Thermodynamic parameters for adsorption of Au(III) by NH_2_-ZIF-90.

Δ*H* (J mol^−1^)	Δ*S* (J mol^−1^·K^−1^)	Δ*G* (KJ mol^−1^)			
		293.15 K	308.15 K	323.15 K	R^2^
–37.09	5.952	–2.378	–2.241	–1.822	0.945

**Table 4:** Determination of Au(III) in lake and tap water samples with recoveries (n = 4).

Samples	Added (μg mL^−1^)	Found (μg mL^−1^)	RSD (%)	Recovery (%)
Lake water	0	–	–	–
	1	0.81	5.3	81.0
	2	1.74	4.9	87.0
	3	2.75	4.8	91.6
Tap water	0	–	–	–
	1	0.83	5.8	83.0
	2	1.82	5.0	91.0
	3	2.80	4.9	93.3

## Data Availability

Data are contained within the article and Supporting Information.

## Supplementary Materials

The following supporting information can be downloaded at: https://www.mdpi.com/article/10.3390/molecules30081826/s1. Figure S1. (A) EDS and (B) element mapping images of NH_2_-ZIF-90. Figure S2. Langmuir isotherm model (red curve) and Freundlich isotherm model (blue curve) for adsorption of Au(III) by NH_2_-ZIF-90. Figure S3. (A) XRD patterns and (B) FT-IR spectra of NH_2_-ZIF-90 after adsorption of Au(III). Table S1. The parameters of the Langmuir and Freundlich isotherms for adsorption of Au(III) by NH_2_-ZIF-90.
