# Supplementary material for: Amino-Modified ZIF-90 for Effective Adsorption of Au(III) in Environmental Water
Source: Molecules. 2025 Apr 18;30(8):1826. doi: 10.3390/molecules30081826 (PMC12029406; doi:10.3390/molecules30081826)
Supplement: Supplementary file 1 [file molecules-30-01826-s001.zip › molecules-3444057-supplementary.pdf]

–*Supporting Information*–

Amino-Modified ZIF-90 for Effective Adsorption of Au(III) in Environmental Water

Na Zhou [1], Xueli Wu [1], Shaoxia Wang [2], Jianfei Qu [2], Yang Tan [1], Chuanlei Luan [1], Xiuli Yin [1], Xuran Wu [2] and Xuming Zhuang [2,*]

1. Yantai Institute of Coastal Zone Research, Chinese Academy of Sciences, Yantai 264003, China; nzhou@yic.ac.cn (N.Z.); xlwu@yic.ac.cn (X.W.); ytan@yic.ac.cn (Y.T.); clluan@yic.ac.cn (C.L.); xlyin@yic.ac.cn (X.Y.)
2. School of Chemistry and Chemical Engineering, Yantai University, Yantai 264005, China; 18865557620@163.com (S.W.); 17860398700@163.com (J.Q.); 201001001732@ytu.edu.cn (X.W.)

* Correspondence: xmzhuang@iccas.ac.cn

(A)

(B)

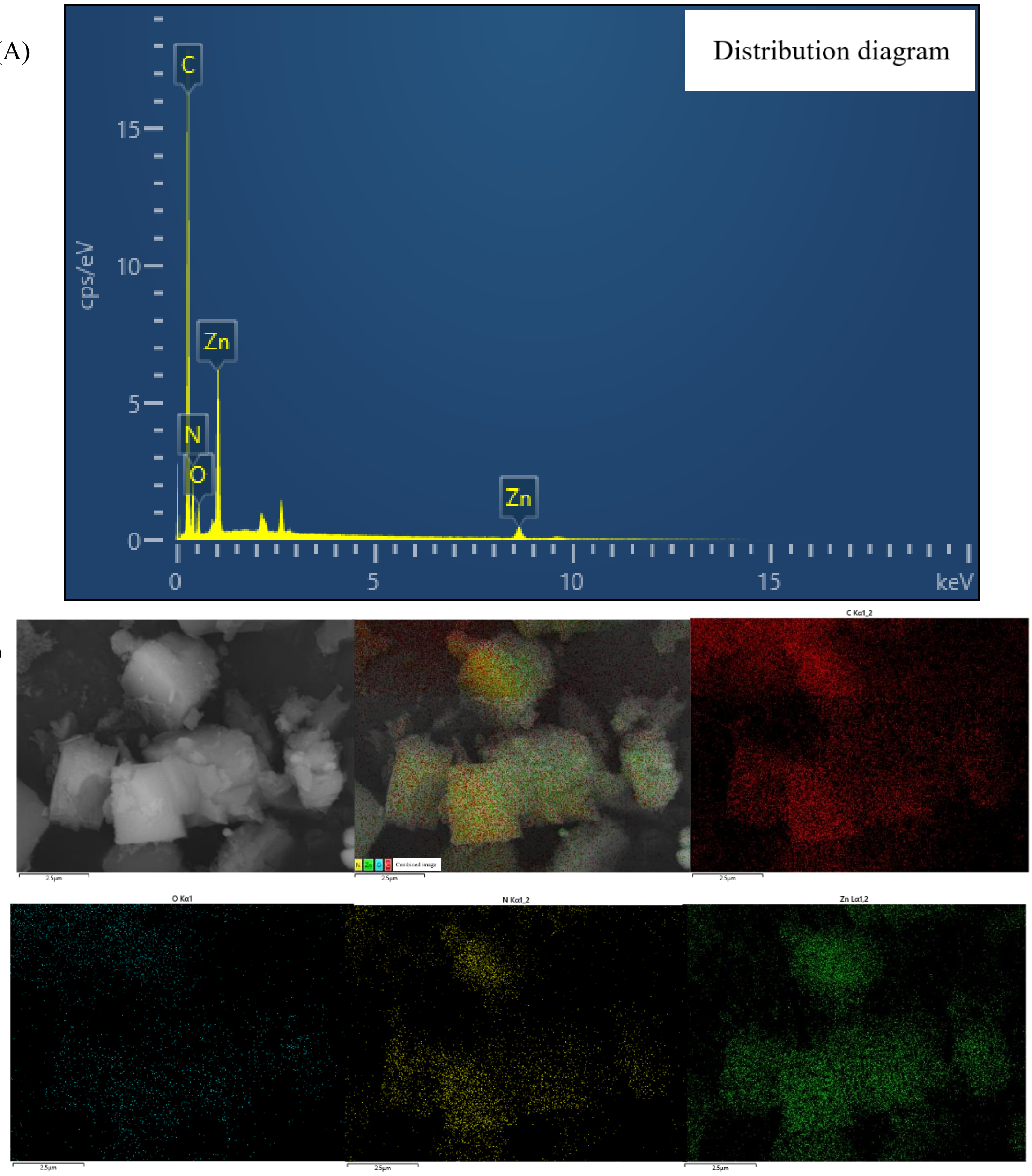

Figure S1. (A) EDS and (B) element mapping images of NH_2-ZIF-90.

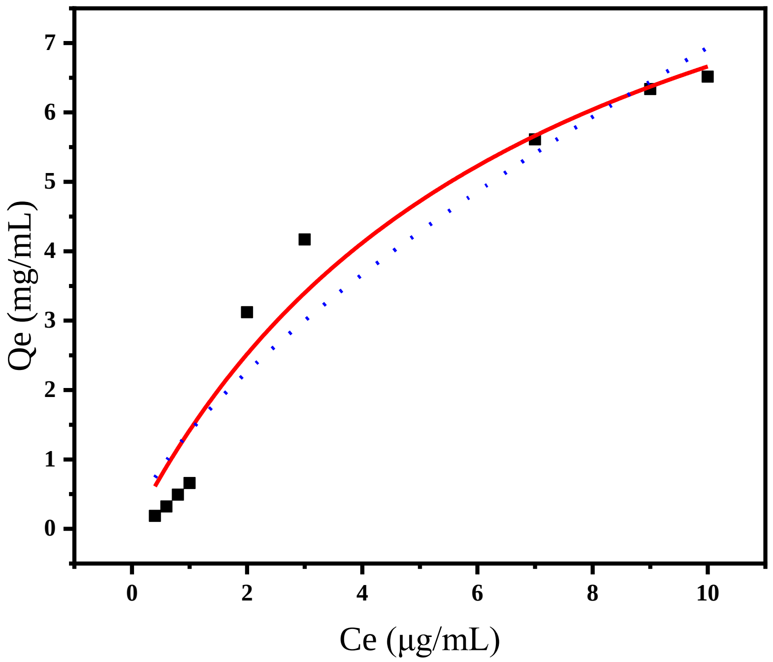

Figure S2. Langmuir isotherm model (red curve) and Freundlich isotherm model (blue curve) for adsorption of Au(III) by NH_2-ZIF-90.

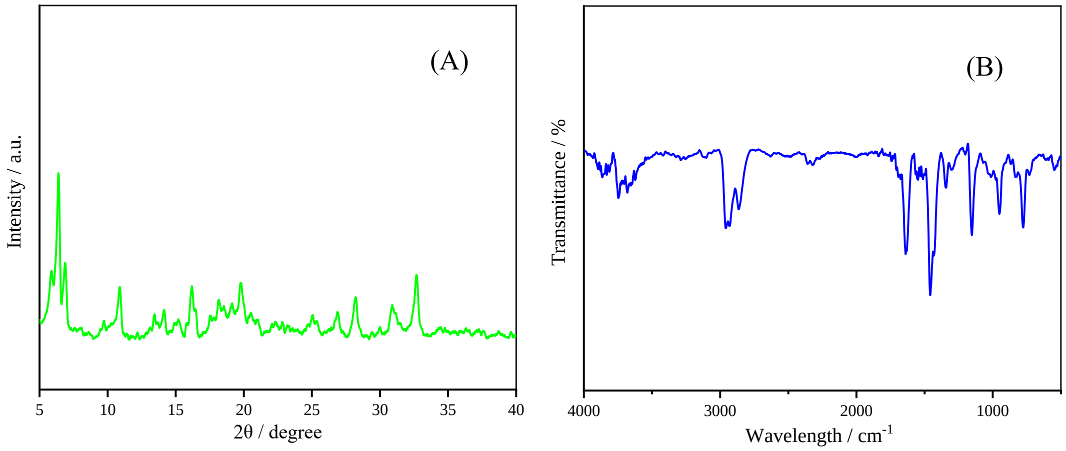

Figure S3. (A) XRD patterns and (B) FT-IR spectra of NH_2-ZIF-90 after adsorption of Au(III).

Table S1. The parameters of the Langmuir and Freundlich isotherms for adsorption of Au(III) by NH_2-ZIF-90.

Langmuir			Freundlich		
Q_m (mg g^{-1})	K_L (L mg^{-1})	R^2	n	K_F (mg g^{-1})	R^2
11.32	0.143	0.951	1.438	1.398	0.918
